# Small Cell Neuroendocrine Carcinoma of the Lung Discovered by Acute Appendicitis Metastasis: An Experience at Our Hospital

**DOI:** 10.7759/cureus.45732

**Published:** 2023-09-21

**Authors:** César J Treviño-Arizmendi, Jorge J Garza-Castillo, Jesús A Saldívar-Rubio, Karla García-Arroyo, Williams L López-Vidal, Julio A Alvarado-Gómez, Luis C Lozano-Carrillo, Grecia C Nery-López, David H Martínez-Puente, Gerardo E Muñoz-Maldonado

**Affiliations:** 1 General Surgery, "Dr. José Eleuterio Gonzalez" University Hospital at the Autonomous University of Nuevo León, Monterrey, MEX; 2 Emergency, "Dr. José Eleuterio Gonzalez" University Hospital at the Autonomous University of Nuevo León, Monterrey, MEX; 3 Histology, Faculty of Medicine, "Dr. José Eleuterio Gonzalez" University Hospital at the Autonomous University of Nuevo León, Monterrey, MEX; 4 Physiology, Biophysics, and Neurosciences, Center for Research and Advanced Studies of the National Polytechnic Institute, Mexico City, MEX

**Keywords:** neuroedocrine neoplasm, symptoms, acute appendicitis, metastatic carcinoma, appendectomy

## Abstract

Acute appendicitis is the most common cause of abdominal pain that requires surgery. Appendiceal cancer is rare, comprising nearly 4% of all gastrointestinal diagnoses. It is common to find neuroendocrine neoplasms due to metastasis in this site. Appendix tumors are usually asymptomatic; however, if they are advanced or have metastases, they can cause abdominal symptoms. Computed tomography (CT) is commonly used to diagnose acute appendicitis in these cases. CT usually shows an increased appendiceal diameter with thickening (>3 mm) of the appendiceal wall, an intraluminal fluid depth >2.6 mm, and periappendiceal inflammation. Histopathological findings confirm the diagnosis. Medical and surgical management depends on physical characteristics such as size, location, and degree of evolution.

We present the case of a 77-year-old woman with a family history of well-controlled type 2 diabetes mellitus and hypertension. She was referred to our institution after four days of abdominal pain in the epigastrium and both flanks accompanied by fever. An abdominal CT showed left pleural effusion and appendicular thickening. Laboratory tests showed high blood glucose levels, leukocytosis at the expense of neutrophils, an increased platelet count, and decreased albumin and total proteins. The CT scan also showed a calcified granuloma in the anterior segment of the right upper lobe and an irregular image with partially defined hypodense borders in the liver in segment IVb.

We report our experience with the diagnosis, management, and treatment decisions of this case. It is important to mention that the first diagnosis was acute appendicitis. This diagnosis motivated us to seek other symptoms and signs by direct questioning and imaging studies leading us to diagnose metastatic lung cancer.

## Introduction

Acute appendicitis is a rare presentation of appendiceal cancer found in 1% of all appendectomy specimens, and most of the cases are from neuroendocrine neoplasms (NEN). According to WHO classification, NENs are divided into neuroendocrine tumors (NET), neuroendocrine carcinomas (NEC), and mixed neuroendocrine non-neuroendocrine neoplasms (MiNENs) [1].

The former comprises 60%-88% of all appendiceal tumors [2]. Regardless of the location, this neoplasm is generally asymptomatic unless it is locally advanced or has metastases that cause abdominal pain or evidence of intestinal obstruction. It is infrequently suspected when there is a sensation of a mass in the right iliac fossa [3].

Histopathology is the gold standard for diagnosing appendiceal tumors. However, cancer is commonly not suspected until the patient presents clinical features and CT findings that suggest acute appendicitis [4].

The type of cancer and its size and location within the appendix determine the surgical treatment of choice, which can range from an appendectomy, either open or by laparoscopy, to right hemicolectomy. In this case, the pathology sample of vermiform cecal appendage reports a small cell NEC. For this patient, besides appendicectomy, palliative systemic treatment was chosen in this clinical case because of the findings on CT and X-ray of the heterogeneous lesion in the upper lobe of the right lung, compatible with the diagnosis of small cell NEC [5].

## Case presentation

The patient was a 77-year-old woman residing in Monterrey, Mexico. Her symptoms started four days before admission with abdominal pain predominantly in the epigastrium and both flanks, accompanied by a fever of 38.2°C. She denied nausea, vomiting, or abnormal bowel movements. She visited a private geriatrician who prescribed a simple abdominal CT scan that showed left pleural effusion and appendicular thickening. The patient was referred to our institution for surgery after receiving antipyretics.

The patient had a family history of death due to acute myocardial infarction and a 25-pack-year smoking history. She also had type 2 diabetes mellitus and hypertension with good control, a surgical history of two cesarean sections 52 years ago, and an open cholecystectomy 32 years ago.

**Figure 1:**
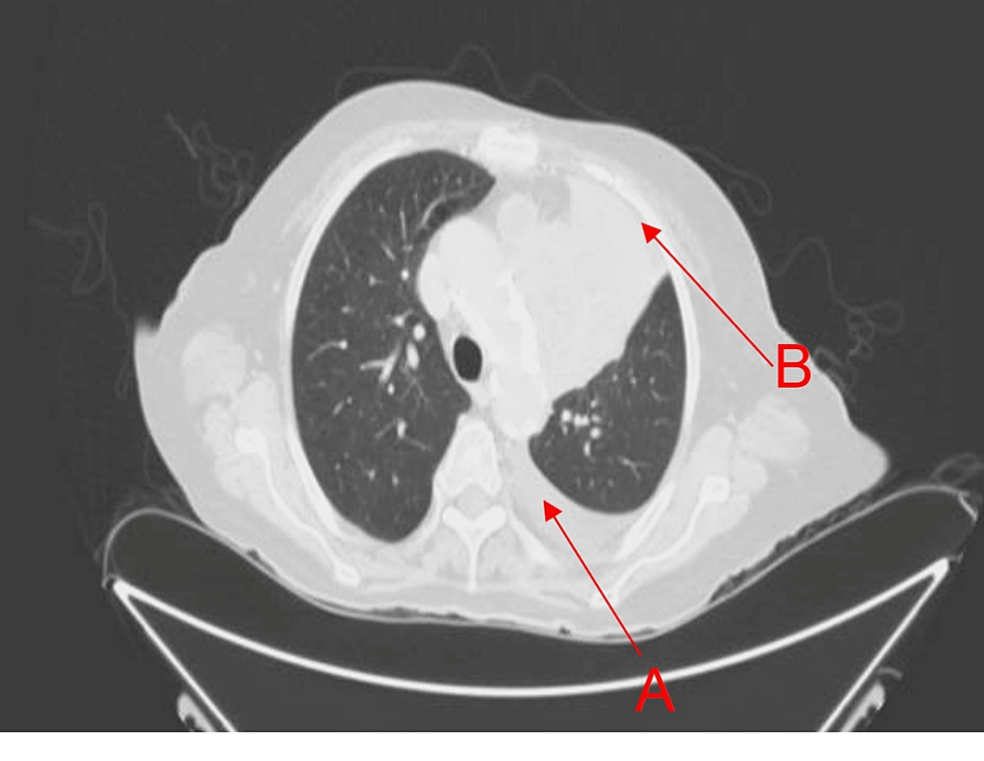
Lung field and airways (A) Atelectasis of the right inferior lobe, secondary to obstruction of the left inferior bronchus, which raises the ipsilateral hemidiaphragm, suggestive of endobronchial neoplasia. (B) Calcified granuloma in the anterior segment of the right upper lobe.

**Figure 2:**
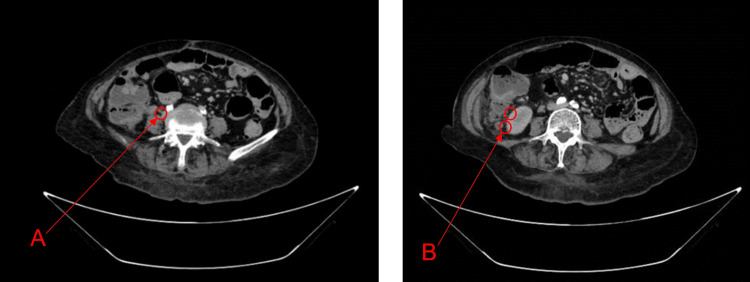
Colonic frame (A) Thickening of the cecal appendix up to 1 cm with wall enhancement after contrast administration, associated with adjacent fat stranding. (B) A greater than 6 mm increase in the transverse diameter with abnormal and heterogeneous wall enhancement.

The patient was afebrile, hemodynamically stable, oriented, and cooperative on arrival. Cardiovascular examination was normal, although decreased vesicular breath sounds were found in the left lung field. The abdomen was soft, with pain on superficial and deep palpation of the right iliac fossa and right flank. Laboratory studies revealed a hemoglobin of 12.5 g/dL, leukocytosis of 14.9 K/uL, platelets of 436 K/uL, blood glucose of 260 mg/dL, blood urea nitrogen (BUN) of 19 mg/dL, serum creatinine of 0.5 mg/dL, and serum albumin of 2.3 g/dL with 5.5 g/dL of total proteins. A contrast-enhanced thoracoabdominal CT was subsequently performed, which revealed a left pleural effusion and a calcified granuloma in the anterior segment of the right upper lobe (Figure 1). An irregular hypodense image with partially defined borders suggestive of metastatic disease was observed in liver segment IVb, and free fluid was observed in the right parietocolic groove in the area of the cecal appendix. The cecal appendix was thickened up to 1 cm with wall enhancement and fat plane effacement associated with acute appendicitis (Figure 2).

An open appendicectomy was performed under balanced general anesthesia. A Rocky-Davis incision was made, and then the tissues were dissected to reach the abdominal cavity. The cecal appendix was explored and identified, and the mesoappendix was dissected. The appendix was cut at its base, ligated with silk 2-0, and the stump was cauterized. A mounted gauze was introduced into the contralateral iliac and pelvic fossa. Hemostasis was confirmed, and the wound was closed by layers at the end of the procedure.

**Figure 3:**
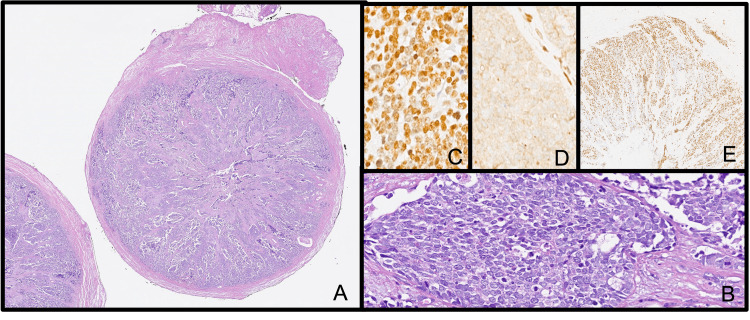
Histopathology samples (A) Cross-section of the cecal appendix, with loss of normal architect and replacement of the lumen by sheets and nests of cells (magnification 1.5x). (B) High magnification of the nests and sheets of ovoid cells with scant cytoplasm; hyperchromatic nuclei with a salt-and-pepper appearance accompanied by apoptotic cells. The stroma presents an Azzopardi effect (20x magnification). (C) Immunohistochemical staining for insulinoma-associated protein 1 (INSM1) with marked and intense nuclear positivity (20x magnification). (D) Immunohistochemical staining for retinoblastoma protein (RB) with loss of nuclear expression and presence of retained internal control. (E) Immunohistochemical staining for Ki67 with an expression greater than 90%. (A) and (B) Conventional hematoxylin and eosin technique; slide digitization with Ventana DP200 Roche Scanner.

The vermiform appendix pathology sample measured 7.1 x .04 x .04 cm with a large tumor diameter of 0.6 cm, with tumor extension to the mucosa, submucosa, muscularis mucosa, muscularis propria, serosa, mesoappendix, and lymphovascular invasion (Figure 3). The diagnosis was a small cell stage 3 neuroendocrine neoplasm. In addition to the appendicectomy, palliative systemic treatment was provided because of the CT findings of a heterogeneous lesion in the upper lobe of the right lung. Afterward, bronchoalveolar lavage (BAL) was performed. A 10-ml sample of cloudy red liquid was obtained from two concentrates, fixed in alcohol, and stained using the Papanicolau technique. The pathology sample was positive, with a few small cell NEC cells [5].

## Discussion

Appendix cancer, an infrequent neoplasia, is generally not considered in the differential diagnosis of a patient with suspected acute appendicitis. Tumors in this region frequently cause symptoms, and they are generally nonspecific when clinical features are present. However, these findings depend on the location of the tumor. Pain in the right iliac fossa commonly occurs with signs of appendicitis when it is located at the base and obstructs the appendicular lumen. However, when the tumor is in the middle region or tip, it normally goes unnoticed, and nonspecific signs or symptoms occur until it is locally advanced or metastatic.

Abdominal pain is the main symptom of acute appendicitis. It can be caused by many pathologies. A differential diagnosis could be intestinal obstruction, intestinal volvulus, or even a primary gastrointestinal tract tumor. Therefore, we used a CT scan to help us identify acute appendicitis and rule out other pathologies.

The primary neoplasms of the appendix are neuroendocrine and non-neuroendocrine. The former comprises two-thirds of appendiceal neoplasms and has the best prognosis. They are commonly diagnosed after surgery based on histopathological findings and sometimes require re-operation of the patient [6].

Definitive treatment depends on several factors, including the histology of the neoplasm. Neuroendocrine neoplasms progress more slowly than other cancers and do not tend to spread. Non-carcinoids tend to have high mitotic rates and spread rapidly.

It is important to understand that surgical intervention depends on the tumor location. A right hemicolectomy is invariably necessary if it is at the base or invades the mesoappendix. However, an appendectomy can be considered in certain cases if it is in the middle portion or the tip [7].

Small cell NEC of the lung has general symptoms, such as weakness, asthenia, weight loss, a cough that worsens or does not go away, or wheezing. Older people may not refer to these symptoms and associate them with aging. Therefore, it is important to assess the patient holistically.

## Conclusions

Appendix cancer, although rare, is relevant. As symptoms are sometimes similar to acute appendicitis, it is difficult to detect, making it an underdiagnosed entity. Nevertheless, the most common cause of appendiceal cancer is neuroendocrine neoplasms, and in this case, it produced a metastatic lung with an NEC. This classification is far more uncommon than a NET; even so, NETs are more indolent incidental lesions. Furthermore, NEC and MiNENs are more aggressive lesions that require a multidisciplinary approach.

This clinical case helps us understand the importance of carrying out a comprehensive patient approach, investigating not only superficial signs and symptoms but also entire systems because, as seen in this case, symptoms of appendicitis can be the first sign reported by a cancer patient. In this case, the relatives and the patient chose palliative measures after the appendectomy. Remember that older patients rarely report their symptoms until the pathology has progressed. In acute appendicitis, it is common for them to come to the emergency department with a perforated appendix. Thus, the importance of this article is to remember a good patient approach in every aspect, from their arrival to the emergency department, during their stay, and during their discharge.
